# Applications of Microarray Technology to Acute Myelogenous Leukemia

**DOI:** 10.4137/cin.s1015

**Published:** 2008-12-22

**Authors:** Rashmi S. Goswami, Mahadeo A. Sukhai, Mariam Thomas, Patricia P. Reis, Suzanne Kamel-Reid

**Affiliations:** 1 Division of Applied Molecular Oncology, Princess Margaret Hospital/the Ontario Cancer Institute, University Health Network, Toronto, ON, Canada; 2 Department of Medical Biophysics, University of Toronto, Toronto, ON, Canada; 3 Department of Laboratory Medicine and Pathobiology, University of Toronto, Toronto, ON, Canada

**Keywords:** acute myelogenous leukemia, gene expression profiling, diagnostics, therapeutics, prognosis, downstream genetic targets

## Abstract

Microarray technology is a powerful tool, which has been applied to further the understanding of gene expression changes in disease. Array technology has been applied to the diagnosis and prognosis of Acute Myelogenous Leukemia (AML). Arrays have also been used extensively in elucidating the mechanism of and predicting therapeutic response in AML, as well as to further define the mechanism of AML pathogenesis. In this review, we discuss the major paradigms of gene expression array analysis, and provide insights into the use of software tools to annotate the array dataset and elucidate deregulated pathways and gene interaction networks. We present the application of gene expression array technology to questions in acute myelogenous leukemia; specifically, disease diagnosis, treatment and prognosis, and disease pathogenesis. Finally, we discuss several new and emerging array technologies, and how they can be further utilized to improve our understanding of AML.

## Introduction

Acute myelogenous leukemia (AML) is a heterogenous disorder characterized by the inhibition of myeloid differentiation in hematopoietic progenitor cells (Estey and Dohner, 2006). This results in the accumulation of relatively undifferentiated “blasts” exhibiting one or more types of early myeloid differentiation within the bone marrow, leading to replacement of normal marrow elements, and clinical aspects of the disease. The most common cause of death is bone marrow failure resulting in anemia, neutropenia, and thrombocytopenia (Estey and Dohner, 2006). Cytokines released by AML blasts also inhibit the differentiation of normal blasts (Youn et al. 2000). Clinically, patients present with fever, fatigue, and spontaneous mucosal and cutaneous bleeding (Aster, 2005). Infections caused by opportunistic organisms such as fungi, *Pseudomonas*, and commensals are frequent (Aster, 2005).

AML is diagnosed based on a combination of morphology, cytochemistry, flow cytometry, and cytogenetics (Aster, 2005; Estey and Dohner, 2006). According to the World Health Organization (WHO) classification, AML can be categorized into four groups: (1) AML with recurrent chromosomal abnormalities; (2) therapy-related AML; (3) myelodysplastic syndrome-associated AML; and, (4) AML, not otherwise specified [NOS] (Harris et al. 1999; Vardiman et al. 2002; Table 1). This scheme attempts to define AML in terms of molecular pathogenesis and outcome, in addition to the criteria listed above. However, heterogeneity still exists within subtypes, especially within the AML, NOS category. Up to 45% of AML patients contain leukemic blasts that do not demonstrate any cytogenetic abnormality (normal karyotype AML) and more accurate methods to determine molecular pathogenesis and clinical outcomes are required for these patients. Molecular analysis of AML through gene expression profiling may help determine patient prognosis and disease pathogenesis.

Global gene expression analysis offers the opportunity to examine the impact of a known disease or disease gene on the genome and transcriptome of the cell. Such an analysis, since the earliest days of its development, has been applied to numerous cancers and other disease systems. For leukemias, associated typically with a single genetic abnormality—usually a single gene mutation (e.g. NPMc, FLT3-ITD, C/EBPα mutation) or a fusion gene arising from a chromosomal translocation (e.g. BCR-ABL, AML1-ETO, X-RARα)—use of global gene expression analysis techniques allows for a deeper understanding of the cellular consequences as well as on the disease as a whole.

We and others have extensively reviewed methods of microarray analysis, study design and interpretation elsewhere (Sultan et al. 2002; Warner et al. 2004). Here, we will discuss the value of analyzing pathways into which deregulated genes may fall, as well as the major uses of array technology in AML. While we focus on using array technology to identify downstream targets of the leukemogenic transcription factors, we will also discuss its potential future applications in the field; namely, in determination of diagnosis, prognosis, and therapeutic response.

## Analysis of Gene Expression Microarrays

### Identifying genes of interest: Biological relevance vs. statistical significance

**Figure 1:**
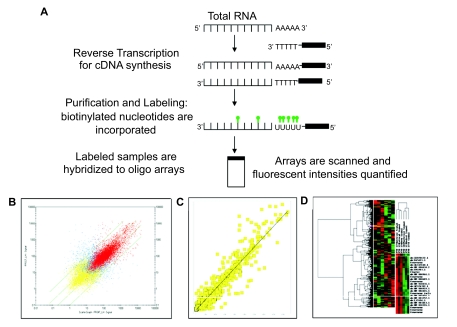
Oligonucleotide microarrays **A)** cDNA synthesis, labeling and hybridization to oligonucleotide array slides. **B**) Correlation coefficient analysis of gene expression data, showing, in red, probes with fluorescent intensities above the threshold of detection, and in yellow, absent fluorescence. **C**) Scatter plot analysis of gene expression data, showing the correlation between two of the samples that clustered together, where most probes have similar expression levels, with some probes differentially expressed between these samples. **D**) Hierarchical clustering of microarray data; in this analysis, samples with similar gene expression profiles are grouped together, cluster of genes is shown on the Y-axis and dendogram or cluster of samples is seen in the X-axis.

A schematic of a typical gene expression array analysis experiment is shown in Figure 1. Different approaches may be used for the interpretation of gene expression array data. One approach relies on the identification of statistically significant deregulated genes between two or more groups of samples. Current analysis of microarray data involves applying both statistical and machine learning techniques, such as hierarchical clustering (Lee et al. 2002), self-organizing maps (Alevizos et al. 2001), or *K*-means clustering (Wang et al. 2000) to organize genes and samples into meaningful groups (Eisen et al. 1998; Vesanto, 1999; Friedman et al. 2000; Sultan et al. 2002; Table 2). These methods have been extensively used in microarray studies (Baugh et al. 2001; Hu et al. 2002; Iscove et al. 2002; Makrigiorgos et al. 2002). There are numerous methods for statistical analysis of microarray data. Most existing tools have been developed for relational types of data, which typically have a large number of instances but low complexity. Thus, high complexity causes many existing tools to fail or provides outcomes with limited usefulness. New tools must be flexible enough to support the diverse tasks associated with clinically relevant genomic research.

While a group of genes may be deregulated in a statistically significant manner in a disease state, there is no assurance that these genes are functionally relevant in the disease. Conversely, in heterogeneous samples, where a mixed population of cells is analyzed as a whole, biologically relevant deregulated genes may not attain statistical significance. For example, in the study conducted by Valk et al. (2004), they identified 16 groups of AMLs with distinct molecular signatures, associated with known and previously-unidentified disease subtypes. However, these studies demonstrated only an association; further analyses are needed to identify biological relevance, especially when working with sets of heterogeneous samples, which may lead to masking of relevant gene expression differences. Such further analysis may yield a set of genes, which, as a group, are biologically significant in the context of the disease. Thus, an in-depth knowledge of the disease or system under investigation is needed.

Pathways analysis is an emerging method by which gene expression array data can be used to give rise to networks of biologically relevant genes. Several pathways analysis tools are available online or commercially, which can aid in the analysis of coordinately deregulated pathways. Others have been developed by indiviual investigators to meet their specific needs. Some tools classify genes based on their Gene Ontology (GO) annotations, while others may map genes into canonical pathways. Still others attempt to define interaction networks or functional relations among gene sets. Tools may also provide the means to compare pathways/interactions among several sets of genes, essentially allowing for a type of comparative analysis to take place. Several tools are available which combine some or all of these functions. All of these tools are based upon curated literature databases. This form of analysis allows the researcher to determine whether a given group of genes, defined within a particular annotation set, is enriched within the dataset, in comparison to the whole genome. The development of interaction networks allows for the identification of sets of genes, not within the same canonical pathway, which may still be involved in the same functional interaction (e.g. interaction networks often help define regulatory interactions, either *via* transcription or protein-protein interaction). Such analyses are useful in rapidly classifying deregulated genes into pathways and interaction networks for validation and functional analysis.

The following discussion further explores the practical use of expression profiling in the clinical setting of acute myelogenous leukemia, paying special respect to the entity of acute promyelocytic leukemia.

## Application of Gene Expression Microarray Technology to Acute Myelogenous Leukemia

Gene expression array technology has been put to a number of uses in order to more fully elucidate AML biology. Broadly, array analysis has been applied to the diagnosis and prognosis of AML; development and understanding of AML therapies; and elucidating the mechanisms of AML pathogenesis (summarized in Table 3). Below, we present case studies of the use of array technology in each of these areas of AML biology.

### Array technology in diagnosis and prognosis

Gene expression profiling has demonstrated diagnostic utility within the research setting. Expression signatures may have predictive power in classifying leukemias from patient samples. Expression profiling requires a large quantity (>10 μg) of high-quality RNA. While it may not replace molecular and cytogenetic testing as a diagnostic method, it is a potentially powerful tool in predicting patient response to therapy, although this area has not yet been extensively explored.

Prediction of known AML subclasses can be performed using gene expression profiling, and AML subgroups with prognostically relevant chromosomal abnormalities can be predicted using this technique (Bullinger and Valk, 2005). The determination of novel AML subclasses has been performed using microarray technology. Bullinger et al. (2004) used cDNA microarrays to determine gene expression in blood and bone marrow samples from 116 AML patients, including 45 patients with normal karyotype AML. This group identified two novel subgroups of AML consisting of patients with normal karyotypes with significant differences in survival times (Bullinger et al. 2004). Unsupervised hierarchical clustering was performed on results from a test set of 59 patients to obtain a set of molecular subgroups with distinct gene expression signatures, and to create a supervised learning algorithm. This algorithm was used to obtain a 133-gene clinical outcome predictor which was then validated on the remaining 57 patients in order to predict overall survival in this group. Using this predictor, overall survival was predicted accurately within the validation group including the subgroup of patients with normal karyotype AML. The gene expression predictor was a strong independent prognostic factor in multivariate analysis (Bullinger et al. 2004).

A second study performed by Valk et al. (2004) determined gene expression profiles within blood or bone marrow of 285 patients with AML. Using unsupervised cluster analysis, sixteen groups of patients with separate molecular signatures were identified. Clustering was driven mainly by chromosomal abnormalities, (i.e. t(8; 21), inv(16), t(15;17), 11q23, –7q), genetic mutations, (i.e. *FLT3*ITD, *FLT3*TKD, N-*RAS*, K-*RAS*, *CEBPA*), and abnormal oncogene expression, (*EVI1*). Novel subclasses of AML, containing normal karyotype AML samples, were obtained, and one cluster with a distinctive gene expression signature associated with significantly poor outcome was identified (Valk et al. 2004). Interestingly, this cluster contained a heterogenous group of samples with regard to known poor-risk markers. The authors suggest that this group may all share a yet unknown biochemical pathway leading to poor prognosis and that this group shares a molecular signature similar to that of normal hematopoietic stem cells (Valk et al. 2004).

Since these two studies, a third group has attempted to independently validate the findings of Bullinger et al. (2004) using the expression signature identified by the latter. Although this group used a different expression array platform, they were able to confirm the prognostic utility of the gene expression signature obtained by Bullinger et al. (2004) (Radmacher et al. 2006). Thus, microarray technology, although currently not in use in the clinical setting may prove to be an important tool in the future in order to further subclassify the disease and help determine patient prognosis and potentially allow clinicians to tailor treatments to individual patients.

Several studies have reported that acute promyelocytic leukemia (APL) has a molecular “signature” associated with the presence of the PML-RARα gene fusion, which is distinct from the signatures of AMLs expressing AML1-ETO, CBFβ-SMMHC or C/EBPα mutations. AMLs expressing NPMc have a gene expression profile (Alcalay et al. 2005), which is also distinct from those of other AMLs. Likewise, other forms of leukemia have distinct gene expression profiles that can be classified based on the kind of leukemia being examined. For example, large scale gene expression profiling has also been used to attempt clinical diagnosis of *de novo* versus MDS-related AML of the M2 subtype, by identifying gene expression signatures associated with these two forms of AML (Oshima et al. 2003).

Interestingly, expression profiling of APL and its microgranular variant (AML M3 and M3v) demonstrated that there are distinct differences between these two forms of promyelocytic leukemia (Haferlach et al. 2005). Additionally, FLT3-ITD is associated with 147 distinct gene expression changes in APL; differentially expressed genes are associated with pathways involving cytoskeletal organization, cell adhesion and migration, coagulation, inflammation, differentiation and myeloid granules (Marasca et al. 2006).

The presence or absence of FLT3 mutations may help determine the prognosis of APL patients. Although many mutations have been identified, the majority, present in approximately 25% of patients, are internal tandem duplications (ITDs). These are known to lead to in-frame insertions within the juxtamembrane region of the receptor. Other less frequent mutations involve the region encoding the activation loop, and most commonly affect codons aspartate 835 and isoleucine 836 (D835/I836). These have been reported in approximately 8% of patients with AML (Gilliland and Griffin, 2002; Kottaridis et al. 2003; Levis and Small, 2003; Stirewalt and Radich, 2003).

A study of 203 patients with PML-RARα-positive APL demonstrated that patients with FLT3 ITDs or D835/I836 mutations had associated poor prognostic indicators. For instance patients with either FLT3 ITDs or activation loop mutations had higher white blood cell counts at presentation, often 10 × 10^9^ cells/L or greater (Gale et al. 2005). The same study discovered that FLT3 ITDs were correlated with M3v subtype, bcr3 *PML* break-point, and expression of reciprocal *RARA-PML* transcripts. Patients with mutant FLT3 had a higher rate of induction death, but no significant difference in relapse or overall survival at 5 years. Microarray analysis revealed differences in expression profiles among patients with FLT3/ITD, D835/I836, and wild-type FLT3 (Gale et al. 2005). The microarray portion of the study revealed that gene expression between FLT3 wild-type and mutant samples was distinct enough that they could be separated into two different clusters. Samples with FLT3 ITD were associated with upregulation of 64 probe sets, including genes involved in or predicted to be involved in, cell cycle control and cell growth (e.g. *SOCS2*, *FRP1*, *PLAGL1*, *TTK*, *CDC16*, *APOBEC3B*) or RNA processing (e.g. *GEMIN4*, *HNRPH1*, *DHX15*). Nineteen probe sets were downregulated in the same population, 5 of which were HLA class 1 genes (*HLA-B71*, allele A*2711, HLA-Cw*1701, *HLA-J*, *HLA-G2.2*). This seems to indicate that the presence of FLT3 mutations have differing effects on gene expression in patients with a t(15; 17) abnormality and they are distinct from expression patterns in FLT3 WT samples (Gale et al. 2005). The discovery that FLT3 mutations are associated with upregulation of genes involved in cell proliferation lends support to the hypothesis that specific mutations providing a proliferative/survival signal cooperate with the PML-RARα—induced differentiation block in APL (Gale et al. 2005; Deguchi and Gilliland, 2002). Thus, although microarrays are currently not being used in the diagnosis of APL, they provide insights into the biology of the disease.

These studies highlight the potential of using gene expression profiling of patients to classify their leukemia and mutation status. However, several important considerations need to be taken into account when interpreting molecular signatures of AMLs. First, different AMLs can arise from different myeloid lineages, or their blasts may resemble more or less differentiated progenitors. Thus, without appropriate lineage and differentiation stage-specific controls, it is difficult to determine whether molecular signatures of different AMLs are due to the disease itself, or merely artifacts of the cell type that predominates in the leukemia. It is also worthwhile to note that molecular signatures in and of themselves do not prove function. For example, while leukemic blasts may exhibit “stem cell-like” signatures, they are not stem cells, nor may they functionally behave like stem cells. Such inferences lie outside the scope of the array data, and require functional studies to more fully elucidate. In interpreting array data, one must always be cautious of the difference between statistical significance and biological relevance of genes in the dataset. Finally, array analysis of AML subtypes to yield molecular signatures of disease allows us to identify potential biomarkers of specific leukemias. These genes are associated with the leukemia, but may not be causative, and therefore part of its molecular mechanism. The application of array technology to this latter question is addressed in more detail below.

### Array technology in the treatment of AML

In addition to a number of studies that utilize microarray technology to subclassify AML and more generally acute leukemias (Kohlmann et al. 2003; Tsutsumi et al. 2004; Song et al. 2006a), this technology has also been used extensively to identify prognostic determinants in AML patients, as well as to better understand the molecular basis of response to therapeutic agents in AML.

Using a cohort of 76 AML patients, one study was able to identify gene expression changes that correlated with response to chemotherapy, and thus was predictive of chemosensitivity (Song et al. 2006b). Another study used a cell line model of doxorubicin resistance to identify gene deregulation patterns in chemoresistant cell lines (Tagliafico et al. 2006). In a similar manner, Tagliafico et al. used gene expression analysis to determine a molecular signature that is predictive for sensitivity to induction of differentiation by retinoids. A comparable analysis was performed to identify prognostic groups using 54 cases of pediatric AML (Yagi et al. 2003). The authors of this study reported a set of 35 genes that included cell cycle and apoptosis regulators that provides prognostic information in pediatric AML.

Array technology has also been applied to gain a better understanding of the molecular basis of therapeutic agents commonly used in AML. This is the case for the drug tipifarnib, a farnesyl transferase inhibitor originally developed to target oncogenic RAS, and shown to be effective in treatment of refractory and relapsed acute leukemias. One study identified genes and genetic pathways that respond to treatment with tipifarnib, and revealed the presence of additional targets in the cell, in addition to RAS (Raponi et al. 2004). To further understand the molecular basis of differentiation therapy using all-*trans* retinoic acid (ATRA) in APL, one study used cDNA microarrays to identify gene expression changes in a time course analysis of ATRA treatment of the NB4 APL cell line model. The study identified a number of genes that were upregulated after ATRA treatment, the majority of which were involved in pathways regulating cellular differentiation, transcription, programmed cell death, and cytokine and chemokine signalling (Yang et al. 2003). Another high-throughput study combined the use of oligonucleotide microarrays and 2D gel difference electrophoresis (2D DIGE) and mass spectrometry to identify both genomic as well as proteomic targets of ATRA in NB4 cells (Wang et al. 2004). Many, but, significantly, not all of these were also attributable to the wild-type retinoic acid response (Liu et al. 2000).

### Array technology in the definition of the mechanism of AML pathogenesis

#### Downstream Targets of AML-Associated Fusion Genes

Array technology has been applied to an understanding of the downstream targets of AML-associated fusion proteins (e.g. X-RARα in acute promyelocytic leukemia, or the t(8; 21) and inv(16) translocation products). Many of these studies are comparative in nature, as researchers aim to understand the signaling and genetic networks commonly deregulated in AML. Several studies have compared the APL fusion proteins PML-RARα with PLZF-RARα, as well as the t(8; 21) product AML1-ETO.

In *in vitro* studies conducted in U937 cells expressing PML-RARα and PLZF-RARα, a comparison of the effects of these two fusions on gene expression was undertaken (Park et al. 2003). In this analysis, deregulated expression of a number of genes identified to be involved in tumor necrosis factor (TNF) α signaling was observed. Strikingly, an independent study examining the effect of ATRA on gene expression in ATRA-sensitive and—resistant NB4 cells also identified TNF-response genes as being induced in response to retinoic acid treatment (Witcher et al. 2003; Witcher et al. 2004). In studies designed to follow up on these latter observations, it was demonstrated that TNFα treatment could cooperate with ATRA in the differentiation of APL cells, implying a potential role for this combination in treating human patients.

Concomitant with the studies described above, others investigated the commonalities among PML-RARα, PLZF-RARα and AML1-ETO, also in U937 cells (Alcalay et al. 2003). Genes deregulated in the presence of all three fusions were found in pathways involved in stem cell maintenance and in the regulation of DNA repair. These observations suggested that AML fusions induced a “de-differentiated” state in leukemic blasts, thus providing for their self-renewal capabilities. In addition, the loss of DNA repair regulation allowed for the accumulation of additional genetic changes, which could accelerate leukemic progression. This is consistent with the observation that external irradiation potentiated the leukemic phenotype in *mCG-PML-RAR*α mice (Walter et al. 2004).

Comparative analysis also identified *MNK1* as over-expressed in U937 cells expressing PML-RARα, PLZF-RARα, and AML1-ETO, and post-translationally stabilized by PML-RARα. (Worch et al. 2004) *MNK1* is functionally associated with eIF4E, and its role in carcinogenesis, as over-expression of *MNK1* leads to phosphorylation of eIF4E, and its subsequent functional activation. Finally, another study examining the effects of PML-RARα, PLZF-RARα and AML1-ETO on U937 cells identified the deregulation of Wnt/β-Catenin signaling common among all three fusions (Muller-Tidow et al. 2004). The Wnt signaling pathway is involved in cellular proliferation, cell-to-cell signaling, and is implicated in the self-renewal of stem cells, suggesting that AML fusions may induce abnormal cellular proliferation in part through deregulated Wnt signaling. Concomitantly, AML-associated translocation products (including PML-RARα) increase the expression of γ-Catenin by activating its promoter region. Increased γ-Catenin expression leads to increased replating efficiency of HSC (Zheng et al. 2004).

Microarray studies have focused on the identification of commonly- and specifically-regulated genes in related leukemias. Both the t(8; 21) and inv(16) forms of AML create fusion proteins involving components of the Core Binding Factor complex, AML1 and CBFβ. A recent study has demonstrated that an overlap exists between the gene expression profiles of t(8; 21)-expressing patient blasts, as compared to inv(16)-expressing blasts. As expected, there are also genes specifically regulated by one fusion, but not the other, in this dataset (Ichikawa et al. 2006).

Studies aimed at examining the downstream targets of the t(8; 21) translocation product AML1-ETO, as well as the naturally-occurring leukemogenic splice variant AML1-ETO9a, identified CD44 as a transcriptional target. Interestingly, CD44 was found to be induced at the mRNA and protein levels, and both variant forms of AML1-ETO were found to bind the CD44 promoter. The authors of this study also identified a number of other genes differentially regulated by AML1-ETO9a. Interestingly, consistent with analysis of the APL fusion proteins, more genes were found to be over-expressed in the presence of this fusion than down-regulated (238 over-expressed >2-fold; 183 under-expressed >2-fold). When considering genes deregulated by >3.5-fold, the contrast was even more striking: 75 genes were over-expressed; 24 were under-expressed (Peterson et al. 2007). It is interesting to consider the implications of this research in the context of the currently accepted models of action for leukemogenic transcriptional repressors such as AML1-ETO and X-RARα. Particularly, why do putative transcriptional repressors have so many over-expressed target genes? Are they simply easier to detect in array and PCR validation studies? Or are they not direct targets of the transcription factors, but are instead secondary or even tertiary effects? Or are they due to some as-yet-unidentified gain-of-function properties of these fusion proteins? As yet, the answer remains unclear.

#### Gene Expression Changes Associated with Other Leukemogenic Mutations

Microarray analysis has also been used to assess the consequences of FLT3 mutation on AML cells (Tickenbrock et al. 2005). Such analysis led to the identification of Frizzled 4, a receptor for Wnt ligands, as being up-regulated in 32D cells expressing FLT3-ITD. Further studies demonstrated a functional link and possible cooperation between FLT3-ITD and Wnt signaling in myeloid transformation. Similar studies identified the proto-oncogene Pim-1 as being up-regulated in the presence of FLT3-ITD (Kim et al. 2005). Additional studies demonstrated that FLT3 mutations activate transcriptional programs that may partially mimic IL-3 activity in cells (Mizuki et al. 2003). Other FLT3-ITD target genes are involved in IL-3-independent pathways that antagonize differentiation.

HOXA9 is frequently over-expressed in AML, suggesting that it may contribute to leukemogenesis. Indeed, forced expression of HOXA9 in mice is leukemogenic. The authors’ data suggested that HOXA9 functions as a cell-type and context-dependent transcriptional activator or repressor, and that its target genes are involved in proliferation or myeloid differentiation. Expression of 14 HOXA9 target genes correlated with its over-expression in AML samples (Dorsam et al. 2004).

#### Array Analysis of Epigenetic Regulation in AML

In a screen to identify methylation targets in AML, the authors of one study identified C/EBPδ as a putative tumor suppressor that is hypermethylated and under-expressed in >35% of AML patients. C/EBPδ was also found to be growth-inhibitory in primary progenitor cells, as well as in FLT3-ITD-transformed cells (Agrawal et al. 2007). Other studies have utilized microarray technology to identify epigenetically silenced genes in AML. Desmond et al. (2007) compared the expression of KG-1 cells,+/− demethylating agents or HDAC inhibitors, to that of similarly-treated primary CD34+ cells. This enabled them to identify genes as epigenetically silenced if they were under-expressed in KG-1, compared to primary CD34+ cells, and over-expressed after drug treatment (Desmond et al. 2007).

#### Array Analysis of Murine Models of Leukemia

Transgenic or transduction/transplant models of AML allow for a unique opportunity to study the effects of leukemogenic mutations *in vivo*. Specifically with respect to APL, only three such studies have been conducted in two mouse model systems: The *mCG-PML-RARα* transgenic line (Walter et al. 2004; Yuan et al. 2007) and the *hCG-NuMA-RARα* line (characterized in Sukhai et al. 2004). In the first set of studies, the authors identified genes deregulated in the presence of PML-RARα in primary bone marrow cultures derived from transgenic mice, and in the context of treatment with external irradiation, in order to measure the accumulation of genetic changes after DNA damage. Further analysis demonstrated a network of deregulated myeloid transcription, postulated to contribute to leukemogenesis in mice. These studies demonstrated that individual PML-RARα^+^ mice had variable gene expression profiles, suggesting that no single, unifying cooperating downstream gene expression change may be required for leukemogenesis in these mice. Our own studies on *hCG-NuMA-RARα* mice utilized pathways analysis to assess the identities of possible cooperating signaling networks in APL. While we identified the deregulation of myeloid transcription in our dataset, we also identified several epigenetic regulators and modulators of apoptosis and cytokine signaling to be deregulated in the presence of NuMA-RARα. Our studies were the first *in vivo* report of gene expression analysis of a variant APL fusion gene.

### Considerations in interpreting array studies

In reviewing array studies of AML, several considerations important to the analysis and interpretation of published array studies become evident. First among these is the nature of the system being studied (human patients, cell line models, or animal models). Cell lines are the most tractable system, often readily available, easily grown and easily manipulated. However, cell lines are immortalized systems with their own stable of genetic changes—some of which may render the cell line genomically unstable. U937 cells, often used for the study of leukemogenic transcription factors, carry 58 chromosomes with multiple genomic changes. The patient-derived APL cell line NB4 carries 78 chromosomes. Additionally, the nature of the manipulation must be taken into consideration when assessing cell line array data: Was the line transduced with a viral vector, or transfected with a plasmid? If transfected, does the plasmid integrate into the genome? Was the transfection transient or stable? If transduced, what was the nature of the viral vector? Did it integrate into the genome? If so, where? What is the dosage of the exogenously introduced gene?

Animal models are more physiologically relevant systems than cell lines, although they are more labor intensive to create, maintain and phenotype. In analyzing array data from animal models, particularly from the hematopoietic compartment, we must take into consideration the heterogeneity of this environment, as well as the temporal and spatial control of the introduced genetic change. It is important to also consider, when comparing across models, whether animals were transgenic, knock-out, knock-in, conditional or transplant/xenograft models in nature.

Human patient samples are perhaps the most ideal system in which to study genetic changes in leukemias. However, as noted previously, they cannot be used to study the mechanism of the disease. Here, too, it is important to recognize the heterogeneity of the bone marrow environment and the nature of the appropriate controls for the array experiment.

Additional considerations in interpreting array studies of AML include the sample size and number of replicates in the study; the need for RNA and protein validation studies as well as functional follow-up or demonstration of clinical relevance; and the purpose of the experiment, as well as the appropriateness of the system being used. For example, one cannot use human samples to identify direct targets of leukemogenic transcription factors. However, while cell lines and animal models may be used for this, as discussed previously, human patient samples must ultimately be used for validation purposes. These questions remain applicable, even when we consider the new and emerging array technologies that are the focus of the final section of this review.

## New and Emerging Array Technologies

### Array comparative genomic hybridization (aCGH) in AML

Array-comparative genomic hybridization, in conjunction with gene expression profiling, has been used to identify gene expression signatures in AML with complex karyotypes (Lindvall et al. 2004; Schoch et al. 2005; Rucker et al. 2006). This approach is valuable in that it allows for the comparison of identified regions of genomic gain and loss with gene expression data, as we would expect that genes situated in regions where the genomic copy number has been altered would either be over- or under-expressed, depending on the change. Such a comparison allows for the screening of array data for gene expression changes that are associated with genomic copy number difference. This permits the identification of genes that are more likely to be directly deregulated by the leukemogenic mutation of interest. Array cGH has also been used to identify genomic imbalances in complex karyotype AML (Rucker et al. 2006), and although not in use diagnostically, can lend insights into disease pathogenesis. Although this technique has some limitations (e.g. it does not allow for the detection of balanced translocations or insertions), novel recurring imbalances were identified in AML patients. Rucker et al. (2006) noted that genomic losses were more common than gains with an increased frequency of 5q, 17p and 7q deletions. Genomic losses involving *TP53* were found in up to 55% of patients, which the authors propose as a mechanism for the genomic instability seen in cases with complex karyotypes. Gains involving 8q and 11q were also noted, and when correlated with gene expression profile data, demonstrated overexpression of genes such as *MYC*, *NSE2*, and *TRIB1*. This study demonstrates that array cGH can be used as a tool to elucidate novel genes involved in the pathogenesis of AML.

### Chip-on-chip technology: Global analysis of DNA binding profiles

Chromatin immunoprecipitation on a chip (ChIP-on-Chip) is a technique that utilizes chromatin immunoprecipitation and microarray analysis to identify protein-DNA interactions in living cells. These studies are useful to researchers studying AML-associated transcription factors, such as AML1-ETO and X-RARα, as they enable analysis of the sequences directly bound by these proteins on the DNA. Thus, regulatory regions, and hence, genes, that are directly transcriptionally modulated by these proteins can be identified.

**Figure 2:**
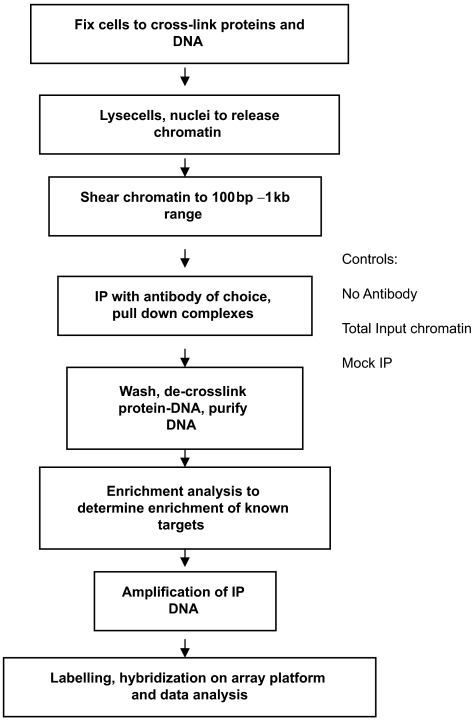
A ChIP-on-Chip workflow. Each step of the chromatin immunoprecipitation stage is optimized for every cell and tissue type. Enrichment analysis to determine successful immunoprecipitation is performed using quantitative real time PCR using primers against target DNA sequences known to be bound by the protein of interest. Large scale genome binding analyses are dependent on the array platform used in the study—these can include promoter arrays, whole genome tiling arrays, or custom made targeted tiling arrays.

A summary of the ChIP-on-chip methodology is presented in Figure 2. Proteins are first fixed to capture cellular protein-DNA interactions by chemical fixation methods. Cells are then lysed, DNA fragmented and immunoprecipitated using antibodies against the target proteins. DNA associated with the targeted protein will be co-purified. The enriched DNA fragments are then labeled and applied to DNA microarrays to detect the enriched signals. Using a number of computational and bioinformatics analysis, a map of protein-genome occupancy is generated. Hybridized sequences can be annotated *in silico* using publicly available tools (including NCBI: www.ncbi.nlm.nih.gov/BLAST and the Transcription Element Search System software package [TESS]: http://www.cbil.upenn.edu/tess/) to determine the characteristics of identified sequences: promoter vs. enhancer/repressor function, as well as possible transcription factor binding sites recognized by the protein of interest. A number of array platforms are currently available for the hybridization of immunoprecipitated DNA. These include printed PCR amplicon arrays, and other commercial oligonucleotide arrays. The choice of array platform depends on the need for high performance, ease of use, cost, and resolution. Whole-genome oligonucleotide tiling arrays currently offer the highest resolution power with probes tiled at approximately 35-base pair intervals. These probes are selected at defined intervals through both coding and non-coding sequences of the entire genome. Base pair resolution describes the density of coverage of the genome on the arrays. High-resolution arrays allow for the more accurate detection of protein/DNA interaction. Other arrays that are commonly used in ChIP-on-Chip assays include promoter arrays, which contain sequences tiled though several thousand promoter regions, and CpG island arrays. In addition to determining transcription factor binding sites, ChIP-on-chip technology is also being applied to examine histone and DNA modifications including methylation, and acetylation, and distributions of chromatin modifying proteins, as well as interactions between proteins and RNA.

ChIP-on-chip technology has been used elsewhere to study the DNA binding sites of the transcription factor E2F1, and thereby identify genes that it directly regulates (Bieda et al. 2006). This analysis revealed binding of E2F1 to novel promoter elements that do not contain known consensus binding sites of E2F1. Another study identified genes directly regulated by the ZNF217 oncogene, proposed to have an important role in neoplastic transformation (Krig et al. 2007). To better understand the pathogenesis of diffuse large cell B lymphomas (DLBCLs) which often exhibit deregulated BCL6 expression, ChIP-on-chip studies have focused on determining direct BCL-6 target genes, and demonstrate that these targets involved in BCL-6 regulated pathways were indeed deregulated in some DLBCLs (Polo et al. 2007). Very recently, a global analysis of genomic DNA binding profiles of the PML-RARα and PLZF-RARα APL fusion proteins have been reported (Hoemme et al. 2008). The screen focusing on PML-RARα identified 372 direct genomic targets of the fusion which include transcriptional modulators as well as cell cycle and apoptosis regulatory genes. Such analysis, in concert with gene expression data analysis, performed for PLZF-RARα, can lend powerful insight into the molecular effects of aberrant fusion proteins within the leukemic cell.

### Use of protein and microRNA microarrays in AML biology

High throughput methods are also being developed to study the proteome and to examine transcriptional regulation through the use of protein and microRNA (miRNA) microarrays. These are novel techniques that show promise in reducing the labour-intensive nature of studying protein and mRNA regulation by allowing the study of complex mixtures of proteins or miRNA simultaneously rather than one by one (Hall et al. 2007). A discussion of the principles behind protein and miRNA microarrays follows.

#### Protein Microarrays

Three types of protein microarrays are currently in use: analytical microarrays, functional microarrays and reverse-phase microarrays (Sreekumar et al. 2001; Zhu et al. 2001; Hall et al. 2004; Bertone and Snyder, 2005; Speer et al. 2005; Hall et al. 2007; Table 4). Analytical microarrays are used to measure binding affinities, specificities, and protein expression levels of proteins in a mixture. Functional protein microarrays are used to study the biochemical activities of an entire proteome in a single experiment. The final type of protein microarray, known as a reverse phase protein microarray (RPA), can be used to determine the presence of proteins altered secondary to disease processes (Hall et al. 2007). Although proteomic techniques have been carried out in order to analyze the proteome within APL systems (Harris et al. 2004; Zhang et al. 2005), protein microarrays have yet to be used to accomplish the same. Comparison of the results obtained with traditional proteomics techniques such as 2D-PAGE-MS to those obtained with protein microarrays will be necessary to determine the advantages and disadvantages of each technique.

#### MicroRNA Microarrays

MicroRNAs (miRNAs) are non-coding RNAs, of 18–22 nucleotides in length, involved in regulation of gene expression by either inhibiting mRNA translation or degrading mRNA (DiLeva et al. 2006). miRNAs are involved in numerous important biological processes including development, differentiation, apoptosis, and proliferation (Bartel, 2004; Harfe, 2005; Cummins and Velculescu, 2006) and are believed to have roles in oncogenesis as well. They can act as either tumor suppressors or as oncogenes (Cummins and Velculescu, 2006). Evidence supports a role for miRNAs in hematopoiesis (Chen et al. 2004; Fazi et al. 2005). These studies focused on studying the role of miRNAs using a candidate gene approach, selecting specific target miRNAs for analysis. One of the first high-throughput studies to examine the global miRNA profile of hematopoeitic cells was the report of ATRA induced changes in miRNA expression in the PML-RARA+ APL cell line, NB4 (Garzon et al. 2007). Using a microarray chip consisting of 245 human and mouse miRNA genes, the group identified a list of miRNAs, including *miR-107*, *let-7a-3*, and *miR-223* differentially expressed during ATRA induced differentiation. As with other RNA expression array systems, these data were also validated using a quantitative real time PCR assay as well as northern analysis using the original cell line model, as well as primary APL patient blasts. MicroRNA profiles have also been used to attempt to classify different classes of acute leukemia. Using a bead-based flow cytometric method (Lu et al. 2005) to determine the expression of 435 miRNAs in ALL, and AML patient samples, another study examined miRNA expression signatures to discriminate between the two types of leukemia (Mi et al. 2007). A similar approach was used to determine that distinct miRNA profiles are associated with several different cytogenetic groups in AML using a miRNA microarray platform (Garzon et al. 2008a). In addition these data also suggested a miRNA profile that was correlated with overall and event-free survival in AML patients (Garzon et al. 2008a). Finally, miRNA microarray platforms have also been used to determine the miRNA signatures that distinguish between variants of AML with and without the NPMc mutation, and thereby help understand the biology of these different AMLs (Garzon et al. 2008b). Taken together, whole genome miRNA profiling of acute leukemias shows promise in the fields of understanding leukemia biology, providing additional diagnostic criteria, and perhaps also in determining novel therapeutic targets. Future studies integrating miRNA profiles with transcriptome and proteome information can yield powerful insights into the pathogenesis and treatment of acute leukemias.

## Conclusions and Future Directions: Comparative Analysis of Multiple Forms of Array Data

With the advent of new and emerging microarray technologies, we are rapidly developing the tools whereby we can utilize multiple high-throughput and whole genome approaches to assay the leukemic cell. The ability to compare data across array platforms will be a powerful tool in the analysis of leukemia pathogenesis and in the identification of novel therapeutic targets in AML. Already, studies in solid tumors have demonstrated the validity of comparison of aCGH and gene expression array data, in order to identify genes whose deregulated expression is correlated to gains or losses of chromosomal regions.

In the analysis of transcription factor-based diseases, comparison of ChIP on chip array data with gene expression array data will allow for the identification of genes that are direct downstream targets of transcription factors, thus allowing for an understanding of direct vs. indirect targets of these proteins. Comparison of gene expression and protein expression array data enables an elucidation of transcriptional effects in disease, vs. translational or post-translational effects. This holds great promise in AML, since the pathogenesis of certain subtypes of this disease is orchestrated by aberrant transcription factors. Integrated computational analysis of gene and protein expression data, as well as miRNA profiling data will help us to understand the protein networks that are deregulated by post-transcriptional or translational means. Taken together, these approaches will yield greater insight into the biology of AML, and allow for an understanding of factors influencing AML pathogenesis, prognosis and treatment.

## Figures and Tables

**Table 1:** The World Health Organization (WHO) and French-American-British (FAB) classifications of acute myelogenous leukemias (AML)

WHO Classification	Description	FAB Classification
**I. AML with recurrent genetic abnormalities**	AML with t(8; 21)(q22; q22), (*AML1/ETO*) AML with abnormal bone marrow eosinophils and inv(16)(p13q22) or t(16; 16)(p13)(q22), (*CBFβ/MYH11*) Acute promyelocytic leukemia with t(15; 17)(q22; q12) (*PML-RARα*) and variants AML with 11q23 (*MLL*) abnormalities	M3: Acute promyelocytic leukemia
**II. AML with multilineage dysplasia**	Following myelodysplastic syndrome (MDS) or MDS/myeloproliferative disease (MPD) Without antecedent MDS or MDS/MPD but with dysplasia in at least 50% of cells in 2 or more lineages	
**III. AML and MDS, therapy related**	Alkylating agent or radiation-related type	
	Topoisomerase II inhibitor-related type	
	Others	
**IV. AML, not otherwise classified**	AML, minimally differentiated	M0: Acute undifferentiated leukemia
	AML, without maturation	M1: AML with minimal differentiation
	AML, with maturation	M2: AML with differentiation
	Acute myelomonocytic leukemia	M4: Acute myelomonocytic leukemia
	Acute monoblastic or monocytic leukemia	M5: Acute monoblastic leukemia
	Acute erythroid leukemia	M6: Acute erythroid leukemia
	Acute megakaryocytic leukemia	M7: Acute megakaryocytic leukemia
	Acute basophilic leukemia	
	Acute panmyelosis and myelofibrosis	
	Myeloid sarcoma	

**Table 2:** Brief description of selected computational methods for gene expression data analysis

Computational methods for array data analysis	Basic concept of method	Reference
Hierarchical Clustering	This method is divided into partitive and agglomerative methods. The agglomerative approach is the most commonly used and it provides a compact summarization of the data. Hierarchical clustering is able to find generic relationships between the resulting clusters; it can point to functional relationships between clustered genes, since genes that are co-expressed might be co-regulated. Clusters are subsequently merged to form a tree structure called dendrogram	Eisen et al. 1998
*K*-means Clustering	This is a simple unsupervised learning algorithm that classifies a given dataset through a certain number of clusters; it requires that the researcher determine *K*, which specifies the number of clusters in the data	Sultan et al. 2002
Self-Organizing Maps (SOMs)	It is a neural network algorithm similar to *k*-means clustering. A hexagonal map unit represents each gene selected by this algorithm. It provides an intuitive visualization of the data, where the expression of a gene is associated with a color in the map, thus similar expression profiles correspond to similar colors.	Vesanto, 1999
Bayesian Networks	This method requires the availability of prior distributions on the data. It provides a graphical display of dependence structure between multiple interacting quantities (e.g. interactions between expression levels of different genes)	Friedman et al. 2000

**Table 3:** Uses of gene expression microarray technology in AML.

Application	Examples
Microarrays in Diagnostics	Determination of gene expression signatures for known AML classes Development of clinical outcome gene expression signature Identification of novel classes of AML, based on gene expression signatures Classification of additional mutation status in patients based on gene expression profiles
Microarrays in Therapeutics	Use of gene expression profiles to predict chemosensitivity Elucidation of the molecular basis of action of AML therapeutic agents
Microarrays in Prognostics	Correlation of gene expression profiles in patients with mutation status and negative prognostic indicators
Microarrays in Understanding the Molecular Basis of Leukemias	Elucidating downstream targets of leukemogenic transcription factors Comparative analysis of downstream targets to identify pathways commonly deregulated in AML Identification of gene expression changes associated with other leukemogenic mutations Correlation between gene expression profiles and epigenetic regulation patterns in AML Molecular characterization of animal models of AML

**Table 4:** Summary of protein microarray technologies. Information adapted from refs (68–73).

Array type	Uses	Technique
Analytical Arrays	Measure: Binding affinities Specificities Protein expression levels (e.g. healthy vs. diseased tissues)	Library of antibodies, aptamers, or affibodies is fixed to a glass slide. Array probed with a protein solution. Detect by labelling protein probes with either fluorescent, affinity, photochemical, or radioisotope tags.
Functional Arrays	Study the biochemical activities and interactions of an entire proteome: Protein-protein Protein-DNA Protein-RNA Protein-phospholipid Protein-small molecule	Composed of full-length functional proteins or protein domains Array probed with a solution containing a specific molecule of interest. Detect by labelling protein probes with either fluorescent, affinity, photochemical, or radioisotope tags.
Reverse-phase Arrays	Determine presence of proteins altered secondary to disease processes (e.g. changes in post-translational modifications)	Cells isolated from various tissues of interest and lysed Lysate arrayed onto a nitrocellulose slide. Slides probed with antibodies against target protein. Antibodies detected with chemiluminescent, fluorescent, or colorimetric assays.
